# Adaptive nonsingular terminal sliding mode control of robot manipulator based on contour error compensation

**DOI:** 10.1038/s41598-023-27633-0

**Published:** 2023-01-06

**Authors:** Zhu Dachang, Huang Pengcheng, Du Baolin, Zhu Puchen

**Affiliations:** 1 School of Mechanical and Electrical Engineering, Guangzhou University, Guangzhou, China; 2 Multi-Scale Medical Robotics Center Limited, Hong Kong, China

**Keywords:** Engineering, Electrical and electronic engineering

## Abstract

To achieve accurate contour tracking of robotic manipulators with system uncertainties, external disturbance and actuator faults, a cross-coupling contour adaptive nonsingular terminal sliding mode control (CCCANTSMC) is proposed. A nonsingular terminal sliding mode manifold is developed which eliminates the singularity completely. In order to avoid the demand of the prior knowledge of system uncertainties, external disturbance and actuator faults in practical applications, an adaptive tuning approach is proposed. The stability of the proposed control strategy is demonstrated by the finite-time stability theory. Then, the developed controller combines adaptive nonlinear terminal sliding mode control (ANTSMC) of joint trajectory tracking and proportion–differentiation control of end-effector contour tracking by introducing the coupling factor between multiple axes based on Jacobian. Moreover, a unified framework of cross-coupling contour compensation and reference position pre-compensation is built. Finally, numerical simulation and experimental results validate the effectiveness of the proposed control strategy.

## Introduction

In the past decades, with the rapid development of modern industrial technology, robotic manipulators have been widely used in machining, laser cutting, welding, and other fields^1^. The accuracy of contour error is one of the key concerns to ensure the quality of machined parts^2^. Contour error is defined as the shortest distance between the current position and the desired contour curve. However, most current control strategy of robotic manipulators focusses on optimizing the tracking performance of each joint to improve the contour machining accuracy of the end-effector^3–6^. Unfortunately, the joint tracking of high accuracy of robotic manipulators cannot effectively solve the problem of contour matching accuracy of the end-effector^6^. One of the main reasons is the lack of coordination of the robotic manipulator^7^. An integrated control strategy of cross-coupling contour error compensation based on chord error constraint, which consists of a cross-coupling controller and an improved position error compensator, was proposed by Zhang et al.^8^. Kommaneesang et al.^9^ pointed that only a few researchers concentrate on solving the contouring problem in the robotic machining system, and the contouring control problem was transformed into the regulation problem by using the method of equivalent errors. Moreover, contour accuracy control was studied in many literatures^10–12^.

SMC is a particular and powerful class of variable structure control essentially which can dynamically adjust based on the current state of the system^13–16^. Thus, the system is forced to track a pre-determined trajectory of sliding mode states. The sliding mode manifold can be designed independent of object parameters and perturbations^17^. Aksu^18^ proposed SMC based on a linear sliding mode manifold which guaranteed that the system states asymptotically converge on the equilibrium point. Inducting a nonlinear term in the linear SMC, Lafrnejani^19^ proposed the terminal SMC (TSMC) to ensure global finite-time stability. Su^20^ proposed an integral sliding mode manifold and its TSMC, and manifested the global finite-time convergence of both sliding mode manifold and tracking error. The above SMC provide an effective and stable control strategy for nonlinear systems, but these rely on the prior knowledge of system uncertainties^21–24^. Besides, these SMC trajectory tracking control of the robotic manipulator only ensures the tracking accuracy of each joint, but the accuracy of contour error is not guaranteed^25,26^. In addition, there exists a singularity phenomenon near the equilibrium point caused by the negative exponent of the TSMC^27,28^. The prior information of system uncertainties is difficult to obtain in practical tasks, such as random fault parameters and disturbances^29^. Considering the uncertainty and control system stability analysis methods^30^, a cross-coupling contour adaptive nonsingular TSMC strategy is proposed to solve the strong coupling contour error problem and avoid the prior information of system uncertainties, external disturbances and actuator faults. Compared to the existing cross-coupling control of robot manipulators, the primary contributions of this paper are summarized as follows:

1. Considering the strong coupling between the contour error and the joint error, the coupling factor with multiple axes based on the Jacobian matrix is proposed. Compared with the method of equivalent errors in Ref.^9^, the accuracy of joint error and contour error is guaranteed simultaneously. Furthermore, a unified framework of cross-coupling contour compensation and reference position precompensation is built.
2. Different from the singularity is generally solved by the parameters in equivalent control law, the uncertainties of the system and the function of the actuator faults are combined with a lumped function, and an adaptive tuning algorithm is adopted to compensate for the lumped uncertainties of the system.
3. Adaptive non-singular terminal sliding mode control with cross-coupled contour is improved, and the stability of the proposed control strategy is demonstrated by the finite-time stability theory. Compared with^20,31^, the tracking errors convergence quickly, and the performance of the proposed control is improved by approximately 61% and 34%, respectively.

This paper is organized as follows: the problem formulation and motivation are indicated in “Problem formulations and motivation” section, ANTSMC for the precise trajectory tracking of the robotic manipulator with dynamic uncertainties, external disturbances and actuator faults is proposed, and its stability with finite-time is discussed in “Adaptive non-singular terminal sliding mode control” section. In “Contour error compensation with cross coupling control” section, the contour error compensation based on the cross-coupling control is presented. Numerical simulation and experiment results are given in “Contour error compensation with cross coupling control” section.

## Problem formulations and motivation

The dynamic of $n -$ DOF (Degree-of-Freedom) robotic manipulator can be expressed by Newton–Euler formula as^32^

1 $$M\left( q \right)\ddot{q} + B\left( {q,\dot{q}} \right)\dot{q} + G\left( q \right) + F\left( {q,\dot{q}} \right) = \tau - \tau_{d} ,$$

where $q,\dot{q},\ddot{q} \in R^{n \times 1}$ are the vectors of position, velocity and acceleration in joint space, respectively. $M\left( q \right) \in R^{n \times n}$ is the positive definite inertial matrix, and $B\left( {q,\dot{q}} \right) \in R^{n \times n}$ is Coriolis and centripetal matrix, and $G\left( q \right) \in R^{n \times 1}$ is the gravity matrix, and $F\left( {q,\dot{q}} \right) \in R^{n \times 1}$ is the vector of the friction, $\tau \in R^{n \times 1}$ is the vector of the input torque, and $\tau_{d} \in R^{n \times 1}$ is the vector of torque with external disturbance.

For actual applications, it is difficult to obtain the precise dynamic model of the robot manipulator as the nonlinearities of the friction and the external disturbances. Hence, (1) can be rewritten as

2 $$M\left( q \right)\ddot{q} + B\left( {q,\dot{q}} \right)\dot{q} + G\left( q \right) + \varphi \left( {q,\dot{q},t} \right) = \tau ,$$

where $\varphi \left( {q,\dot{q},t} \right) \in R^{n \times 1}$ is the lumped uncertainty of the system and can be defined by

3 $$\varphi \left( {q,\dot{q},t} \right) = \Delta M\left( q \right)\ddot{q} + \Delta B\left( {q,\dot{q}} \right)\dot{q} + \Delta G\left( q \right) + \tau_{d} + F\left( {q,\dot{q}} \right),$$

where $\Delta M\left( q \right)$, $\Delta B\left( {q,\dot{q}} \right)$ and $\Delta G\left( q \right)$ are the uncertain parameters un-modeled for the dynamics of robot manipulator.

The properties of (2) are satisfied with

### Property 1

$M\left( q \right)$ is the symmetric and positive matrix, and bounded by

4 $$mI < M\left( q \right) = M^{T} \left( q \right) \le \overline{m}I,$$

where $m$ and $\overline{m}$ are positive constant parameters, respectively, and $0 < m < \overline{m}$. $I \in R^{n \times n}$ is an identity matrix.

### Property 2

$M\left( q \right) - 2B\left( {q,\dot{q}} \right)$ is the skew symmetric matrix and satisfy with

5 $$D^{T} \left[ {M\left( q \right) - 2B\left( {q,\dot{q}} \right)} \right]D = 0,$$

where $D$ is any vector.

### Property 3

$G\left( q \right)$ is bounded by

6 $$\left\| {G\left( q \right)} \right\| \le G_{k} ,$$

where $G_{k} \in R^{n \times 1}$ is positive constant matrix.

Considering the problem of actuator faults during the operating process of the robot manipulator, (2) can be rewritten as follows

7 $$M\left( q \right)\ddot{q} + B\left( {q,\dot{q}} \right)\dot{q} + G\left( q \right) + \varphi \left( {q,\dot{q},t} \right) = \tau + f,$$

where $f = \gamma \left( {t - T_{f} } \right)\phi \left( {q,\dot{q},\tau } \right)$ is the function of the actuator faults, and $\gamma \left( {t - T_{f} } \right) \in R^{n \times n}$ is the time profile of the faults, $\phi \left( {q,\dot{q},\tau } \right) \in R^{n \times 1}$ is the vector of the faults, and ${T}_{f}$ is the time of appearance of the faults.

The time profile of the faults $\gamma \left(t-{T}_{f}\right)$ is a diagonal matrix, yields to

8 $$\gamma \left( {t - T_{f} } \right) = diag\left[ {\gamma_{1} \left( {t - T_{f} } \right),\gamma_{2} \left( {t - T_{f} } \right), \ldots ,\gamma_{n} \left( {t - T_{f} } \right)} \right],$$

where $\gamma_{i}$ denote the influence of the faults to the *i*th state.

The time profile mode of the faults is given by

9 $$\gamma_{i} \left( {t - T_{f} } \right) = \left\{ {\begin{array}{*{20}l} 0 \hfill & {if\;t < T_{f} } \hfill \\ {1 - e^{{ - \sigma_{i} }} \left( {t - T_{f} } \right)} \hfill & {if\;t \ge T_{f} } \hfill \\ \end{array} } \right.,$$

where $\sigma_{i} > 0$ is the evolution rate of the faults.

## Adaptive non-singular terminal sliding mode control

### Adaptive non-singular terminal sliding mode control

The position tracking error denoted by $e\left( t \right) \in R^{n \times 1}$ in joint space is defined as

10 $$e = q - q_{d} ,$$

where $q_{d} \in R^{n \times 1}$ is the desired trajectory.

Non-singular terminal sliding mode manifold $s$ is defined as

11 $$s = \dot{e} + c_{1} e^{\alpha /\beta } + c_{2} e^{\eta } {\text{sgn}} \left( e \right),$$

where $c_{1} ,c_{2} \in R^{n \times n}$ are constant positive definite diagonal matrix, $\alpha$ and $\beta$ are positive odd integers, and satisfy with $1 < \alpha /\beta < 2$, $\eta > 0$. $e^{\eta } {\text{sgn}} \left( e \right) = \left[ {e_{{_{1} }}^{\eta } {\text{sgn}} \left( {e_{1} } \right), \cdots ,e_{n}^{\eta } {\text{sgn}} \left( {e_{n} } \right)} \right]^{T}$. ${\text{sgn}} \left( * \right)$ is the signum function.

Derivation (11) with respect to time, we obtain

12 $$\dot{s} = \ddot{e} + \frac{{c_{1} \alpha }}{\beta }e^{\alpha /\beta - 1} \dot{e} + c_{2} \eta e^{\eta - 1} \dot{e} = M^{ - 1} \left( {\tau + f - \varphi - B\dot{q} - G} \right) - \ddot{q}_{d} + \frac{{c_{1} \alpha }}{\beta }e^{\alpha /\beta - 1} \dot{e} + c_{2} \eta e^{\eta - 1} \dot{e}.$$

The positive-definite Lyapunov function is given by

13 $$V_{1} = \frac{1}{2}s^{T} Ms.$$

Derivation (13) with respect to the time, and combining with (12), yields to

14 $$\dot{V}_{1} = s^{T} M\left( {\ddot{q} - \ddot{q}_{d} } \right) + s^{T} Bs + s^{T} M\left( {\frac{{c_{1} \alpha }}{\beta }e^{{\left( {\alpha /\beta } \right) - 1}} \dot{e} + c_{2} \eta e^{\eta - 1} \dot{e}} \right).$$

Substituting (7) into (14), we obtain

15 $$\dot{V}_{1} = s^{T} M\left[ {M^{ - 1} \left( {\tau - B\dot{q} - G - \varphi + f} \right)} \right] - s^{T} M\ddot{q}_{d} + s^{T} M\left( {\frac{{c_{1} \alpha }}{\beta }e^{\alpha /\beta - 1} \dot{e} + c_{2} \eta e^{\eta - 1} \dot{e}} \right) + s^{T} Bs.$$

Simplified (15), we obtain

16 $$\dot{V}_{1} = s^{T} \left[ {\tau + B\left( {s - \dot{q}} \right) - G - \varphi + f + M\left( {\frac{{c_{1} \alpha }}{\beta }e^{\alpha /\beta - 1} \dot{e} + c_{2} \eta e^{\eta - 1} \dot{e} - \ddot{q}_{d} } \right)} \right].$$

Let $\dot{V}_{1} = 0$, the equivalent control law $\tau_{eq}$ is derived as

17 $$\tau_{eq} = - B\left( {s - \dot{q}} \right) + G + \varphi - f - M\left[ {\frac{{c_{1} \alpha }}{\beta }e^{\alpha /\beta - 1} \dot{e} + c_{2} \eta e^{\eta - 1} \dot{e} - \ddot{q}_{d} } \right].$$

Assumed that the uncertainties and actuator faults of the robot manipulator are defined as

18 $$K = \varphi - f.$$

The upper bound of function $K$ is estimated as follows

19 $$\left\| K \right\|_{\max } = \left\| \varphi \right\|{ + }\left\| f \right\|,$$

where $\left\| * \right\|$ is the standard Euclidean norm.

The switch control law $\tau_{sw}$ is given by

20 $$\tau_{sw} = - kMs\left| s \right| - kM{\text{sgn}} \left( s \right)\left| s \right|^{\mu } ,$$

where $k = \left\| K \right\|_{\max } + \upsilon$*,* $\upsilon$ is the switch control gain, and $\upsilon \ge 0$*,* and $0 < \mu < 1$ is a positive constant.

Thus, the NTSMC is derived as

21 $$\tau = \tau_{eq} + \tau_{sw} = - M\left[ {\frac{{c_{1} \alpha }}{\beta }e^{{{\raise0.7ex\hbox{$\alpha $} \!\mathord{\left/ {\vphantom {\alpha \beta }}\right.\kern-0pt} \!\lower0.7ex\hbox{$\beta $}} - 1}} \dot{e} + c_{2} \eta e^{\eta - 1} \dot{e} - \ddot{q}_{d} } \right] - B\left( {s - \dot{q}} \right) + G + K - M\left( {\left\| K \right\|_{\max } + \upsilon } \right)\left( {s\left| s \right| + {\text{sgn}} \left( s \right)\left| s \right|^{\mu } } \right).$$

Since $\varphi$ and $f$ are the lumped uncertainty of the system and the function of the actuator faults, respectively, $K$ is unknown function caused by $\varphi$ and $f$. However, the design process of NTSMC relies on the value of function $K$, and hence an adaptive algorithm is proposed to determine the value of uncertainties and actuator faults.

The estimated error is defined as

22 $$\tilde{K} = K - \hat{K},$$

where $\hat{K}$ is the estimated value of $K$. Assumed that the uncertainties and actuator faults change slowly, there has $\dot{K} = 0$.

Derivation (22) with respect to the time, we obtain

23 $$\dot{\tilde{K}} = \dot{K} - \dot{\hat{K}} = - \dot{\hat{K}}.$$

The control law $\tau$ of the system can be rewritten as follows:

24 $$\tau = M\left( {\ddot{q}_{d} - \frac{{c_{1} \alpha }}{\beta }e^{{\frac{\alpha }{\beta } - 1}} \dot{e} - c_{2} \eta \left| e \right|^{\eta - 1} \dot{e}} \right) - B\left( {s - \dot{q}} \right) + G + \hat{K} - M\left( {\left\| {\hat{K}} \right\|_{\max } + \upsilon } \right)\left( {s\left| s \right| + {\text{sgn}} \left( s \right)\left| s \right|^{\mu } } \right).$$

The positive definite Lyapunov function with estimated error is given by

25 $$V_{2} = \frac{1}{2}s^{T} s + \frac{1}{2\xi }\tilde{K}^{T} \tilde{K},$$

where $\xi$ is positive integer.

Derivation (25) with respect to the time, yields to

26 $$\dot{V}_{2} = - \left( {\left\| {\hat{K}} \right\|_{\max } + \upsilon } \right)s^{T} \left( {s\left| s \right| + {\text{sgn}} \left( s \right)\left| s \right|^{\mu } } \right) - s^{T} M^{ - 1} \tilde{K} - \frac{1}{\xi }\tilde{K}^{T} \dot{\hat{K}}.$$

Adaptive algorithm is given by

27 $$\dot{\hat{K}} = - \xi \left( {M^{ - 1} } \right)^{T} s.$$

Substituting (27) into (26), we obtain

28 $$\dot{V}_{2} = - \left( {\left\| {\hat{K}} \right\|_{\max } + \upsilon } \right)s^{T} \left( {s\left| s \right| + {\text{sgn}} \left( s \right)\left| s \right|^{\mu } } \right) \le - \left( {\left\| {\hat{K}} \right\|_{\max } + \upsilon } \right)\left( {\eta_{1} \left\| s \right\|^{2} + \eta_{2} \left\| s \right\|^{\mu } } \right) \le 0,$$

where $$\eta_{1} = \frac{1}{n}\sum\limits_{i = 1}^{n} {\left| {s_{i} } \right|}$$, $$\eta_{2} = \left\| s \right\|^{2 - \mu } \left| s \right|_{\max }^{\mu - 1}$$, and $$\left| s \right|_{\max } = \max \left( {\left| {s_{1} } \right|, \cdots ,\left| {s_{n} } \right|} \right)$$.

**Figure 1:**
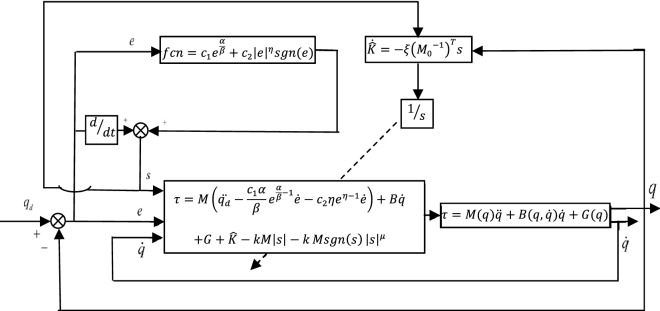
Schematic of the proposed ANTSMC.

The schematic of the proposed ANTSMC is shown in Fig. 1.

### Finite-time stability analysis

#### Theorem 1

For finite-time stability with fast time convergence, the Lyapunov function $V\left( x \right)$ with initial value $V_{0}$ is given by

29 $$\dot{V}\left( x \right) + aV\left( x \right) + bV^{\delta } \left( x \right) \le 0,\;\forall x \ge x_{0} ,\;V\left( {x_{0} } \right) \ge 0,$$

where $a > 0$, $b > 0$, $0 < \delta < 1$, and $V\left( x \right)$ satisfy the inequality at any $x_{0}$.

The stability time of convergence $T$ can be calculated by

30 $$T \le x_{0} + \frac{1}{{a\left( {1 - \delta } \right)}}In\frac{{aV^{1 - \delta } \left( {x_{0} } \right) + b}}{b}.$$

#### Proof

Considering the Lyapunov function as

31 $$V = \frac{1}{2}s^{T} Ms.$$

Derivation (31) with respect to the time, yields to

32 $$\dot{V} = s^{T} M\dot{s} + \frac{1}{2}s^{T} \dot{M}s.$$

Substituting (12) into (32), we obtain

33 $$\dot{V} = s^{T} M\left( {\ddot{e} + \frac{{c_{1} \alpha }}{\beta }e^{{\left( {\alpha /\beta } \right) - 1}} \dot{e} + c_{2} \eta e^{\eta - 1} \dot{e}} \right) + s^{T} Bs = s^{T} \left[ {\left[ {\tau + f - } \right.B\dot{q} - G - \varphi + Bs + M\left( {\ddot{e} + \frac{{c_{1} \alpha }}{\beta }e^{{\left( {\alpha /\beta } \right) - 1}} \dot{e} + c_{2} \eta e^{\eta - 1} \dot{e} - \ddot{q}_{d} } \right)} \right].$$

Substituting (21) into (33), yields to

34 $$\dot{V} = s^{T} \left[ { - \tilde{K} - \hat{k}s\left| s \right| - \hat{k}{\text{sgn}} \left( s \right)\left| s \right|} \right].$$

As $s^{T} \cdot s = \left\| s \right\|^{2}$ and $s^{T} {\text{sgn}} \left( s \right) = \left\| s \right\|$, (34) can be simplified as

35 $$\dot{V} = - \left\| {\tilde{K}} \right\|\left\| s \right\| - \hat{k}\left\| s \right\|^{2} \left\| s \right\| - \hat{k}\left\| s \right\|\left\| s \right\|.$$

Assumed that the uncertainties and the actuator faults changes slowly, there has

36 $$\dot{V} = - \left( {\hat{k}\left\| s \right\|^{2} + \hat{k}\left\| s \right\|} \right)\left\| s \right\| \le - \left( {s^{T} \hat{k}s + s^{T} \hat{k}{\text{sgn}} \left( s \right)} \right)\left\| s \right\|^{\mu } ,$$

where $$\mu \tilde{ = }1$$.

As

37 $$s^{T} Ks^{r} = \sum\limits_{i = 1}^{n} {k_{i} s_{i}^{r + 1} } \ge \gamma \left\{ {\sum\limits_{i = 1}^{n} {\frac{1}{2}\overline{m}s_{i}^{2} } } \right\}^{\lambda } \ge \gamma \left( {\frac{1}{2}s^{T} Ms} \right)^{\lambda } ,$$

where $$\lambda \triangleq \left( {1 + r} \right)/2$$, $$\gamma \triangleq k_{\min } \left\{ {2/m} \right\}^{\lambda }$$, $$k_{\min } \triangleq \min \left( {k_{i} } \right)$$.

Then

38 $$\dot{V} \le - \left\| s \right\|\gamma V - 1^{{\left( {\mu + 1} \right)/2}} \left\| s \right\|\gamma V^{{\left( {\mu + 1} \right)/2}} .$$

According to Theorem 1, the proposed adaptive non-singular terminal sliding mode control is finite time stable.

When $x_{0} = 0$, the convergence time can be expressed as

39 $$t_{s} \le \frac{1}{{\Omega_{1} \left( {1 - \delta } \right)}}In\left( {1 + \frac{{\Omega_{1} V_{0}^{{\left( {1 - \mu } \right)/2}} }}{{\Omega_{2} }}} \right),$$

where $$\Omega_{1} = \left\| s \right\|\lambda_{\max } \left( {\hat{k}} \right)$$, $$\Omega_{2} = 1^{{{\raise0.7ex\hbox{${\left( {\mu + 1} \right)}$} \!\mathord{\left/ {\vphantom {{\left( {\mu + 1} \right)} 2}}\right.\kern-0pt} \!\lower0.7ex\hbox{$2$}}}} \left\| s \right\|\lambda_{\max } \left( {\hat{k}} \right)$$.

## Contour error compensation with cross coupling control

### Contour error of end-effector of robot manipulator

**Figure 2:**
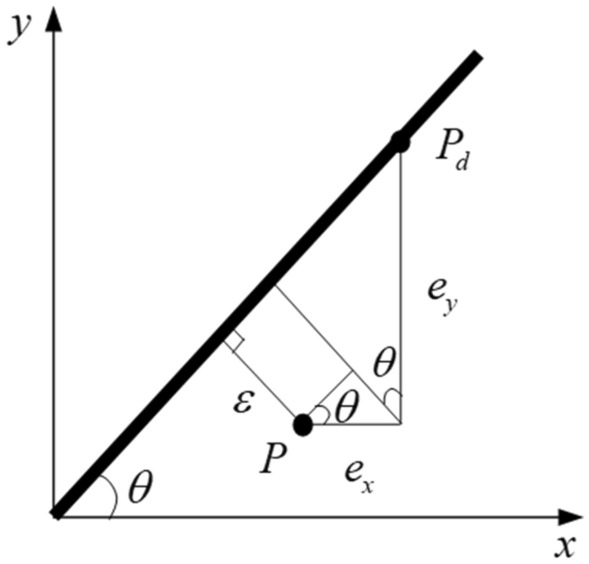
Error model of the straight-line contour.

The trajectory planning interpolation method can be used to fit the trajectory contour into a straight line or a circular contour. The contour error model of the straight-line contour for an XY planar is shown in Fig. 2.

$P$ is the actual position of the end-effector of robotic manipulator, $P_{d}$ is the reference point, $\varepsilon$ is the contour error, $e_{x}$ and $e_{y}$ are the error components along with $x$ and $y$ axis of the tracking error $e$, respectively. $\theta$ is the angle between the reference trajectory $y$ and the $x$ axis.

Assumed that the contour error is the shortest distance between the prevailing position and the desired contour curve. The contour error of plane line is defined as

40 $$\varepsilon = c_{x} e_{x} + c_{y} e_{y} ,$$

where $c_{x} = - \sin \theta$, $c_{y} = \cos \theta$ are the cross coupling operator.

### ANTSMC based on cross coupling

The contour motion control with cross coupling is given by

41 $$\tau_{c} = K_{p} \varepsilon + K_{d} \dot{\varepsilon },$$

where $K_{p}$ is the proportional gain, and $K_{d}$ is the differential gain of the contour motion control.

As compensation of contour and trajectory tracking control are carried out in the task space and in the joint space, respectively, so the mapping relationship between two space is established as follows

42 $$e_{c} = J\left( q \right)e_{q} ,$$

where $e_{c}$ is the vector of contour tracking error in the task space, and $e_{q}$ is the vector of trajectory tracking error in the joint space, $J\left( q \right)$ is the Jacobian matrix.

According to (42), the contour tracking error is derived, yields to

43 $$\varepsilon = \left[ {\begin{array}{*{20}c} {c_{x} } & {c_{y} } \\ \end{array} } \right]e_{c} = \left[ {\begin{array}{*{20}c} {c_{x} } & {c_{y} } \\ \end{array} } \right]J\left( q \right)e_{q} .$$

The rectifier gain of contour tracking error compensation is given by

44 $$C_{n} = \left( {\left[ {\begin{array}{*{20}c} {c_{x} } & {c_{y} } \\ \end{array} } \right]J\left( q \right)} \right)^{T} .$$

Combining (21) with (41), we obtain

45 $$\tau = M\left[ {\ddot{q}_{d} - \frac{{c_{1} \alpha }}{\beta }e^{{\left( {{\raise0.7ex\hbox{$\alpha $} \!\mathord{\left/ {\vphantom {\alpha \beta }}\right.\kern-0pt} \!\lower0.7ex\hbox{$\beta $}}} \right) - 1}} \dot{e} - c_{2} \eta e^{\eta - 1} \dot{e}} \right] - B\left( {s - \dot{q}} \right) + G + \hat{K} - \left( {\left\| {\hat{K}} \right\|_{\max } + \upsilon } \right)\left( {Ms\left| s \right| + M{\text{sgn}} \left( s \right)\left| s \right|^{\mu } } \right) + \left( {K_{p} \varepsilon + K_{d} \dot{\varepsilon }} \right)C_{n} ,$$

where $C_{n} \in R^{n \times 1}$ is the contour error compensation rectifier gain.

#### Remark

From Eq. (45), one can see that the proposed CCCANTSMC is a combination of ANTSMC control for joint trajectory tracking and PD contour control for end-effector. The ANTSMC in joint space ensures the stability of the robotic system, while the PD control in the workspace is used to reduce contour errors. The goal of the proposed cross-coupled controller is to improve the tracking performance of the joint and further improve the contour tracking performance of the end-effector.

**Figure 3:**
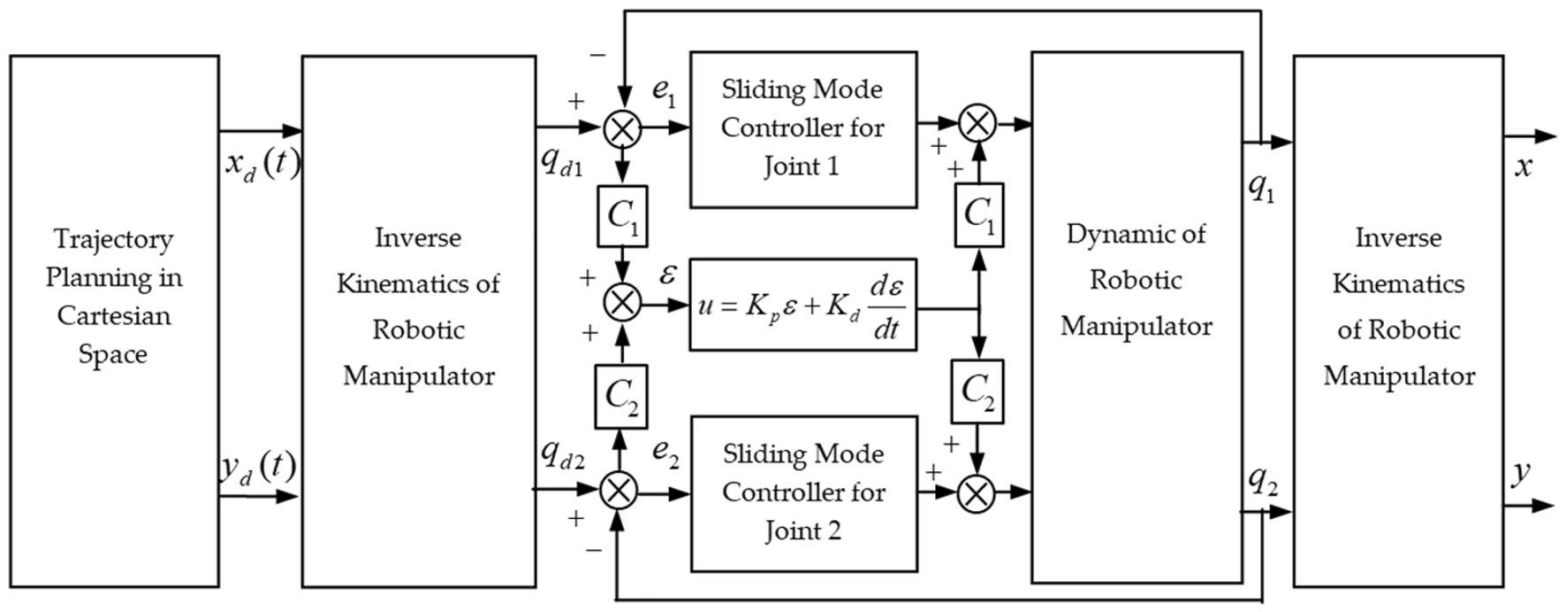
Schematics of ANTSMC with cross-coupling.

The schematic of the ANTSMC with cross-coupling is shown in Fig. 3.

## Experiments

**Figure 4:**
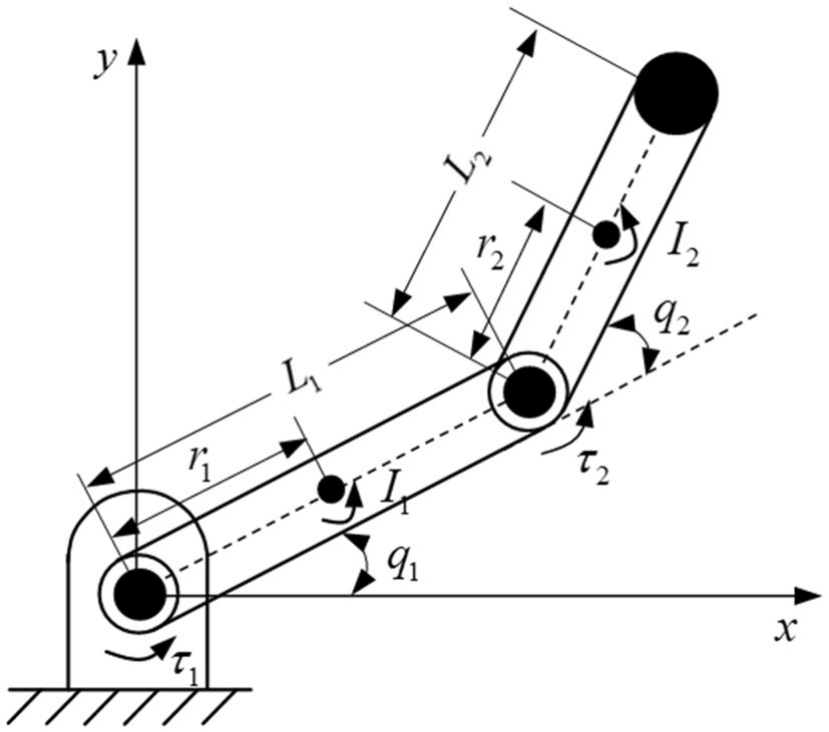
Structure of robot manipulator with two-link.

Robotic manipulator with two-link is used to illustrate the effectiveness of the proposed control strategy, shown as Fig. 4.

Assumed that the mass of each link is concentrated. The dynamic equation of the robotic manipulator with two-link is derived as follows

$$\left[ {\begin{array}{*{20}c} {\tau_{1} } \\ {\tau_{2} } \\ \end{array} } \right] = \left[ {\begin{array}{*{20}l} {M_{11} } \hfill & {M_{12} } \hfill \\ {M_{21} } \hfill & {M_{22} } \hfill \\ \end{array} } \right]\left[ {\begin{array}{*{20}c} {\ddot{q}_{1} } \\ {\ddot{q}_{2} } \\ \end{array} } \right] + \left[ {\begin{array}{*{20}l} {B_{11} } \hfill & {B_{12} } \hfill \\ {B_{21} } \hfill & {B_{22} } \hfill \\ \end{array} } \right]\left[ {\begin{array}{*{20}c} {\dot{q}_{1} } \\ {\dot{q}_{2} } \\ \end{array} } \right] + \left[ {\begin{array}{*{20}c} {G_{1} } \\ {G_{2} } \\ \end{array} } \right],$$

where $$\left[ {\begin{array}{*{20}c} {\tau_{1} } & {\tau_{2} } \\ \end{array} } \right]^{T}$$ is the vector of the input torque, and

$$M_{11} = m_{1} r_{1}^{2} + m_{2} r_{2}^{2} + m_{2} L_{1}^{2} + 2m_{2} L_{1} r_{2} \cos q_{2} + I_{1} + I_{2} ,\;M_{12} = m_{2} r_{2}^{2} + m_{2} L_{1} r_{2} \cos q_{2} + I_{2} ,$$

$$M_{21} = m_{2} r_{2}^{2} + m_{2} L_{1} r_{2} \cos q_{2} + I_{2} ,\;M_{22} = m_{2} r_{2}^{2} + I_{2} ,\;B_{11} = - 2m_{2} L_{1} r_{2} \sin q_{2} \dot{q}_{2} ,$$

$$B_{12} = - 2m_{2} L_{1} r_{2} \sin q_{2} \dot{q}_{2} - 2m_{2} L_{1} r_{2} \sin q_{2} \dot{q}_{1} ,\;B_{21} = - 2m_{2} L_{1} r_{2} \sin q_{2} \dot{q}_{1} ,\;B_{22} = 0,$$

$$G_{2} = m_{2} r_{2} g\cos (q_{1} + q_{2} ),\;G_{1} = \left( {m_{1} r_{1} + m_{2} L_{1} } \right)g\cos q_{1} + m_{2} r_{2} g\cos \left( {q_{1} + q_{2} } \right).$$

$J\left( q \right)$ is the Jacobian matrix which is defined as

$$J\left( q \right) = \left[ {\begin{array}{*{20}l} { - L_{1} \sin \left( {q_{1} } \right) - L_{2} \sin \left( {q_{1} + q_{2} } \right)} \hfill & { - L_{2} \sin \left( {q_{1} + q_{2} } \right)} \hfill \\ {L_{1} \cos \left( {q_{1} } \right) + L_{2} \cos \left( {q_{1} + q_{2} } \right)} \hfill & {L_{2} \cos \left( {q_{1} + q_{2} } \right)} \hfill \\ \end{array} } \right].$$

Considering the influence of uncertainties and the actuator faults of robotic manipulator, $\varphi \left( {q,\dot{q},t} \right)$ and $\phi \left( {q,\dot{q},\tau } \right)$ are selected as follows

$$\phi \left( {q,\dot{q},\tau } \right) = \left\{ {\begin{array}{*{20}l} {30\sin \left( {q_{1} q_{2} } \right) + 4\cos \left( {\dot{q}_{1} q_{2} } \right) + 15\cos \left( {\dot{q}_{1} \dot{q}_{2} } \right)} \hfill & {T_{f1} \ge 1.5} \hfill \\ 0 \hfill & {T_{f2} \ge 1.5} \hfill \\ \end{array} } \right..$$

Set the simulation time is $3\;{\text{s}}$, the sampling step is $0.001\;{\text{s}}$, and the initial state is $$\left[ {\begin{array}{*{20}c} {0.3} & {0.3} \\ \end{array} } \right]^{T}$$, the desired trajectory along with $x$ and $y$ are given by

$$x = \left\{ {\begin{array}{*{20}l} {0.3 - 0.2t,} \hfill & {0 \le t \le 1} \\ {0.1,} & {1 < t \le 2} \\ {0.2\left( {t - 1} \right) - 0.1,} & {2 < t \le 3} \hfill \\ \end{array} } \right.,$$

$$y = \left\{ {\begin{array}{*{20}l} {0.2t + 0.1,} \hfill & {0 \le t \le 1} \hfill \\ {0.3 - 0.2\left( {t - 1} \right),} & {1 < t \le 2} \\ {0.1,} & {2 < t \le 3} \hfill \\ \end{array} } \right..$$

Parameters of traditional PID, ANTSMC and cross coupling control are given by Table 1, respectively.

**Table 1:** Parameters of controller.

Type of controller	Parameters
Traditional PID	$K_{P} = diag\left[ {4500,\,4500} \right]$ $K_{I} = diag\left[ {150,\,150} \right]$ $K_{D} = diag\left[ {650,\,650} \right]$
ANTSMC	$c_{1} = diag\left[ {10,\,10} \right]$, $c_{2} = diag\left[ {10,\,13.5} \right]$ $\eta = diag\left[ {0.5,\,0.5} \right]$, $\alpha = 3$, $\beta = 5$ $\upsilon = diag\left[ {50,\,165} \right]$, $\xi = 10$
ANTSMC with cross coupling	$K_{p} = 500$, $K_{d} = 100$

**Figure 5:**
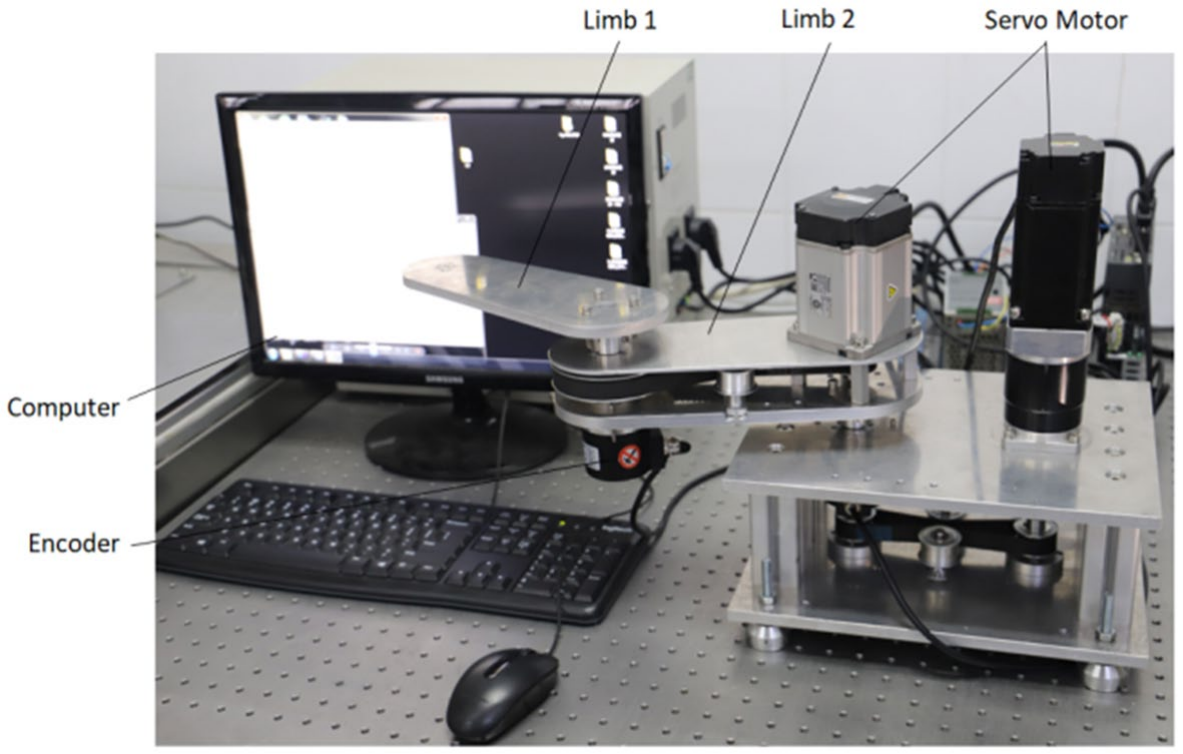
Experiment platform of robot manipulator with two-link.

**Figure 6:**
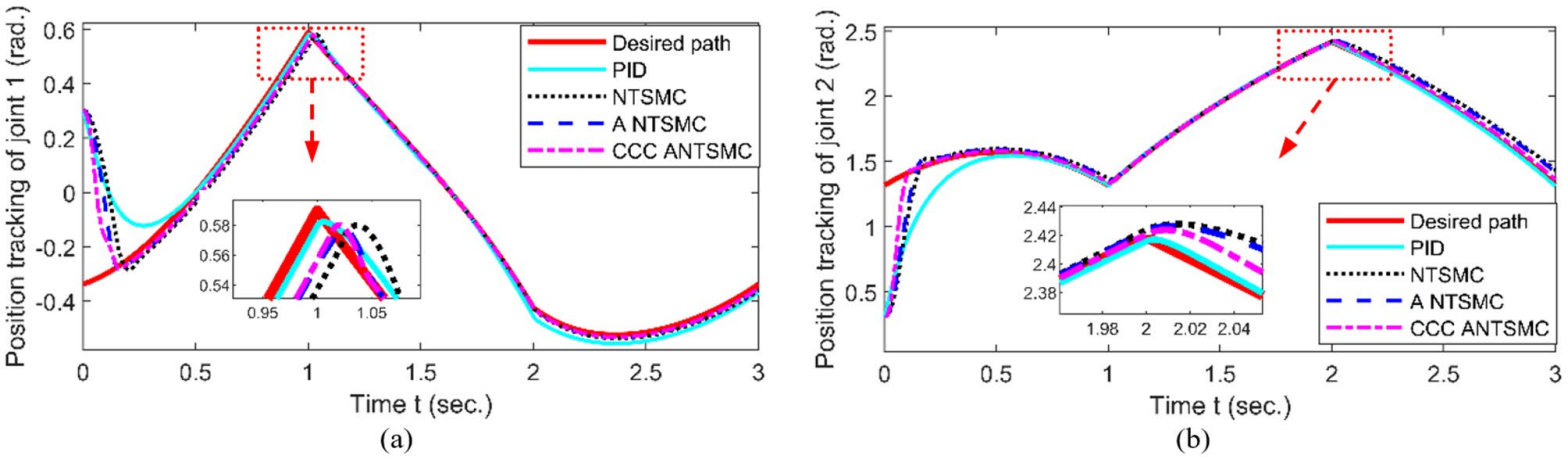
Trajectory tracking in joint space (a. Joint 1#, b. Joint 2#).

**Figure 7:**
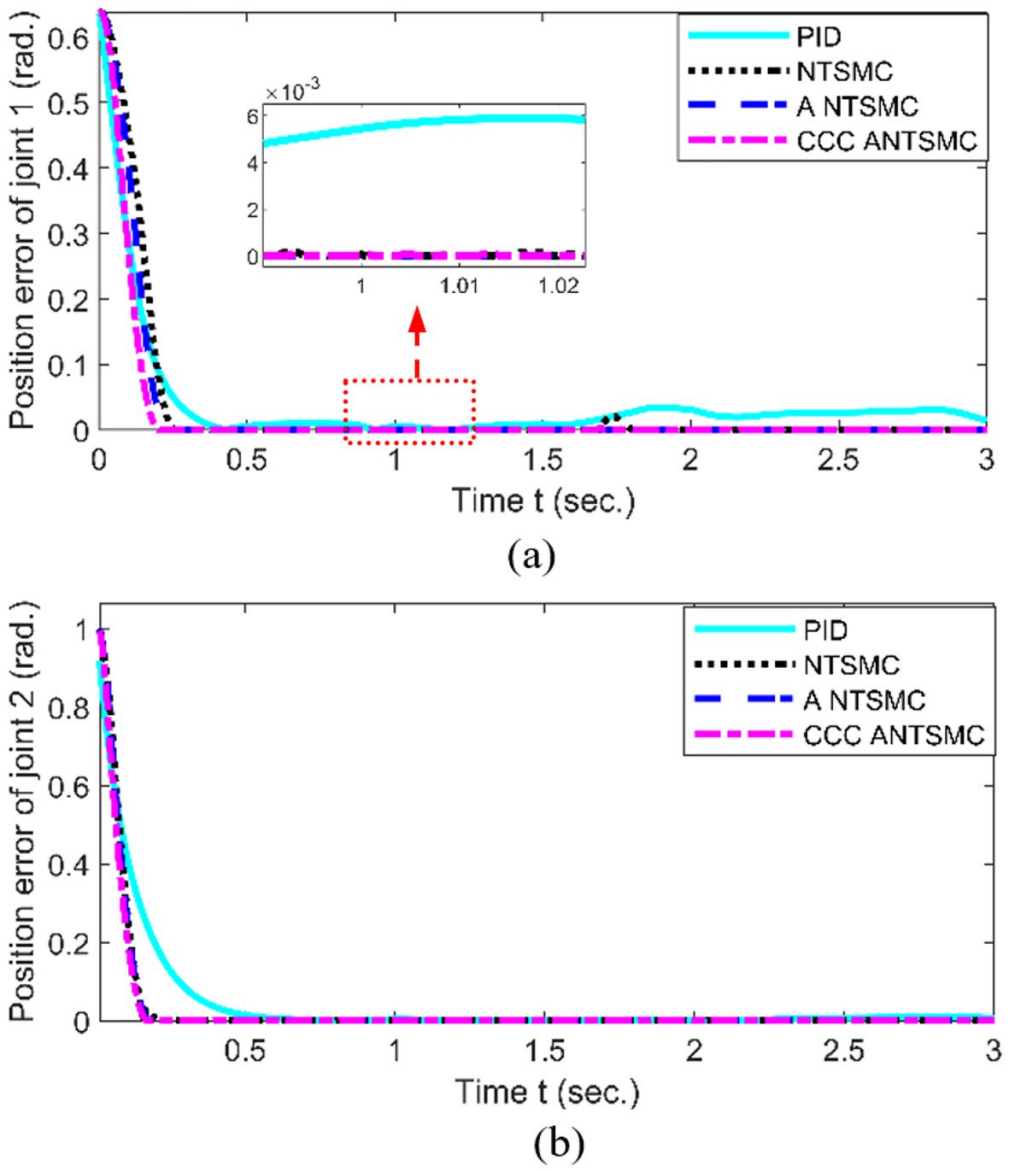
Tracking error (a. Joint 1#, b. Joint 2#).

**Figure 8:**
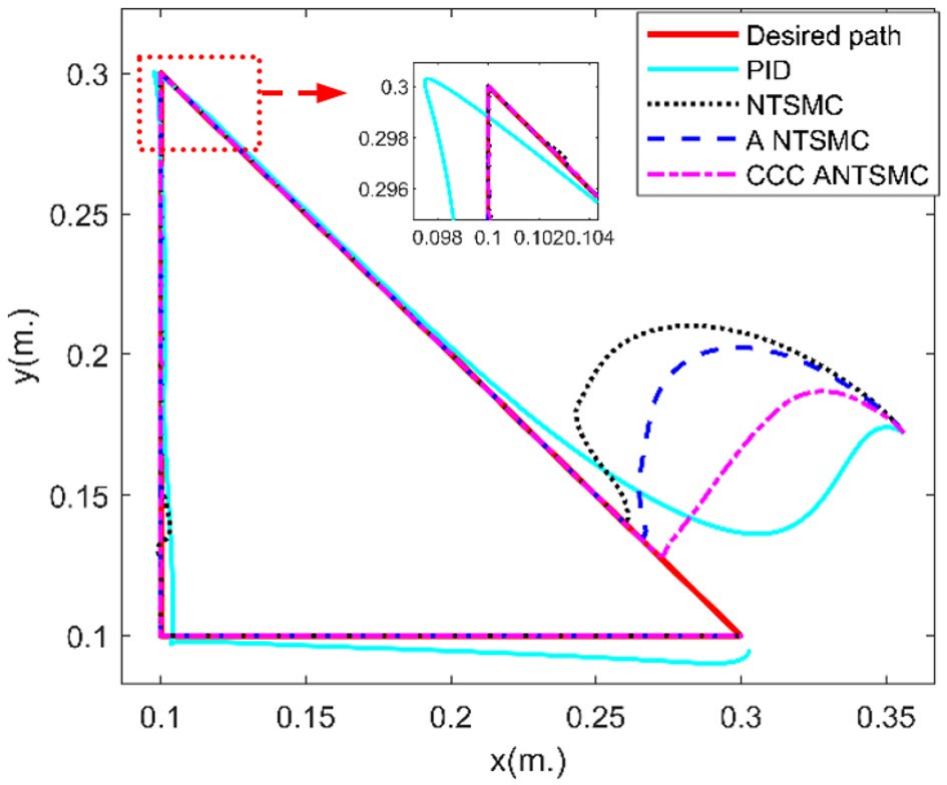
Contour tracking error of the end-effector.

The experiment platform is built, shown as Fig. 5. Trajectory tracking of joint 1# and 2# in the joint space is shown in Fig. 6. The error of trajectory tracking in the joint space are shown in Fig. 7. The contour tracking of the end-effector of the robotic manipulator is shown in Fig. 8.

The mean square value of the tracking error is defined as

46 $$e_{j} = \sqrt {\frac{1}{N}\sum\limits_{i = 1}^{N} {\left( {\left\| {e_{j} (k)} \right\|^{2} } \right)\quad j = 1,2} } ,$$

where $N$ is the number of simulation step.

As shown in Figs. 6 and 7, compared with PID, NTSMC^20^, and ANTSMC^31^, when the system has obvious position errors, the joint tracking errors of the proposed CCCANTSMC reach the convergence more quickly. Moreover, there are the actuator faults after 1.5 s, the control performance of PID controller decreases remarkably, and the control performance of NTSMC converges after a slight fluctuation. However, the NTSMC used adaptive algorithm can ensure the stability of the system effectively. In addition, from Fig. 8, it is obvious to see that compared with PID, NTSMC and ANTSMC, the proposed CCCANTSMC can quickly optimize the contour error when there are significant position errors in the system.

Table 2 shows that the mean squared contour error for PID, NTSMC and ANTSMC is 0.0076 m, 0.0051 m and 0.0044 m, respectively, while the mean squared contour error for CCCANTSMC is 0.0029 m. Compared to PID, NTSMC, and ANTSMC, the control performance of CCCANTSMC is improved by approximately 61%, 43%, and 34%, respectively. The proposed CCCANTSMC significantly reduces the contour error and the systematic tracking error. Moreover, the mean squared tracking error of each joint of CCCANTSMC is smaller than that of PID, NTSMC and ANTSMC. The experimental results demonstrate that the proposed control strategy has better properties than the remaining three control methods in contour control.

**Table 2:** Performance comparison of each controller.

Controller	Average tracking error of joint 1 (rad)	Average tracking error of joint 2 (rad)	Average error of contour (m)
Traditional PID	0.0461	0.0826	0.0076
NTSMC	0.0467	0.0715	0.0051
ANTSMC	0.0387	0.0651	0.0044
CCCANTSMC	0.0297	0.0482	0.0029

In this paper, due to sudden changes in position, velocity and acceleration at the junction inflection point of adjacent straight segments, each controller will suffer performance degradation to a certain extent at the time of 1 s and 2 s, but the adaptive tunning method of the proposed control strategy for this kind of mutation is better than the traditional PID, NTSMC and ANTSMC. However, it is necessary to perform trajectory planning processing at these sudden changes to optimize the controller performance, which will be a desired future research work.

## Conclusions

In this paper, a cross-coupling contour adaptive nonsingular terminal sliding mode control (CCCANTSMC) is proposed for the issue of precise contour tracking of the robotic manipulator in the presence of system uncertainties, external disturbance, and actuator faults. Based on the strengths of the NTSMC for driving the system state to the equilibrium point in finite time, the adaptive tuning approach is proposed. Thus, the prior knowledge of system uncertainties, external disturbance, and actuator faults is avoided and the singularity problem is eliminated. Introducing coupling factors among the multi-axes based on Jacobian, ANTSMC of joint tracking and PD control of end-effector contour tracking is combined to improve the accuracy of contour error. Furthermore, a unified framework of cross-coupling contour compensation and reference position pre-compensation is built. The experimental results are shown to prove the effectiveness of the proposed control strategy.

## Data Availability

The datasets used and/or analysed during the current study available from the corresponding author on reasonable request.
